# Supplementary material for: Realistic prediction and engineering of high-Q modes to implement stable Fano resonances in acoustic devices
Source: Nat Commun. 2023 Oct 27;14:6847. doi: 10.1038/s41467-023-42621-8 (PMC10611717; doi:10.1038/s41467-023-42621-8)
Supplement: Supplementary file 1 — Supplementary Information [file 41467_2023_42621_MOESM1_ESM.pdf]

Realistic prediction and engineering of high-Q modes for the implementation of stable Fano resonances to acoustic devices — Supplementary Information —

Felix Kronowetter[1,2,3], Marcus Maeder[1], Yan Kei Chiang[2], Lujun Huang[2], Johannes D. Schmid[1], Sebastian Oberst[3], David A. Powell[2], and Steffen Marburg[1]

[1] *Chair of Vibro-Acoustics of Vehicles and Machines, Department of Engineering Physics and Computation, Technical University of Munich, TUM School of Engineering and Design*
[2] *School of Engineering and Information Technology, University of New South Wales, Northcott Drive, Canberra, ACT 2600, Australia*
[3] *School of Mechanical and Mechatronic Engineering, Centre for Audio, Acoustics and Vibration, Faculty of Engineering and IT, University of Technology Sydney, Sydney, Australia*

S 1 Coupled mode theory

We use coupled mode theory [1, 2, 3] to predict the location of the BIC. For simplicity, we consider a reduced two-dimensional coupled rectangular waveguide-resonator system shown in Fig. S 1.

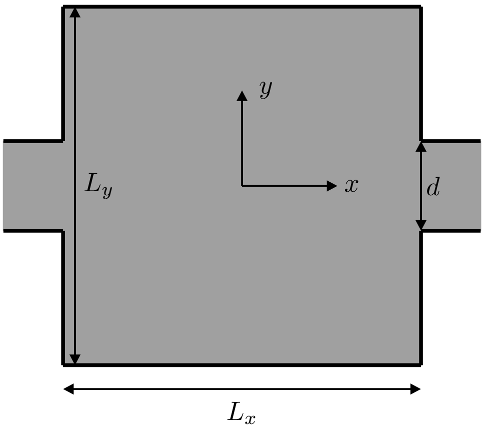

Fig. S 1. Schematic drawing of a coupled two-dimensional waveguide-resonator system.

To make the conclusion as general as possible, we set the width of the waveguide $d = 1$ (unitless), and the width and height of the resonator are L_x and L_y, respectively. Also, the center of the resonator is set as the origin, and the left and right waveguides are attached along the x-axis. Thus, the waveguide spans from $y = -1/2$ to $y = +1/2$. The first step is to compute the eigenfrequencies and eigenmodes of a closed resonator. They eigenfrequencies can be solved analytically with Neumann boundary conditions as follows

$$\frac{\nu_{m,n}^2}{\omega_0^2} = \left(\frac{(m-1)}{L_x}\right)^2 + \left(\frac{(n-1)}{L_y}\right)^2 \quad , \quad n, m = 1, 2, 3, \ldots \tag{1}$$

where $\nu_{m,n}$ is the resonant frequency and $\omega_0 = \pi c/d$, c is the speed of sound in air. We obtain the corresponding modes ψ by

$$\psi_{m,n} = \sqrt{\frac{(2-\delta_m^1)(2-\delta_n^1)}{L_x L_y}} \cos\left(\frac{\pi(m-1)(2x+L_x)}{2L_x}\right) \cos\left(\frac{\pi(n-1)(2y+L_y)}{2L_y}\right) \tag{2}$$

with δ_n^1 and δ_m^1 being the Kronecker delta. The propagating wave numbers in the waveguide are given by

$$\frac{\nu^2}{\omega_0^2} = \frac{(k_p^2}{\pi^2} + (p-1)^2 \tag{3}$$

with k_p being the wavenumber of the pth channel of the waveguide. We obtain the corresponding modes ϕ by

$$\phi_p = \sqrt{(2-\delta_p^1)}\cos\left(\frac{\pi(p-1)(2y+1)}{2}\right)e^{\mathrm{i}k_p x}. \tag{4}$$

Then the coupling matrix elements between eigenmodes of closed resonator and pth propagation channels of the left/right waveguide can be obtained by

$$W_{m,n;p} = \int_{-\frac{1}{2}}^{\frac{1}{2}} \psi_{m,n}(x = -\frac{L_x}{2}, y)\phi_p(x = -\frac{L_x}{2}, y)dy. \tag{5}$$

After obtaining the coupling matrix, we compute the complex eigenvalues of the effective Hamiltonian [4, 5, 6, 7], where the real parts correspond to the resonance frequencies and the imaginary parts to the half resonance linewidth. Thus, the search for BICs amounts to finding the zero imaginary part of the eigenvalues. In general, the eigenfunction of any BIC can be decomposed as

$$\phi_{BIC} = \sum_{m,n} a_{m,n}\psi_{m,n}(x,y). \tag{6}$$

Since the BIC is perfectly decoupled from the continuum, its eigenfunction must be given by

$$\int_{-\frac{1}{2}}^{\frac{1}{2}} \phi_{BIC}(x = -\frac{L_x}{2}, y)dy = 0. \tag{7}$$

When two resonant states approach each other as a function of a certain continuous parameter, interference causes an avoided crossing of the two states in their energy positions. At the same time, one of the resonance line widths vanishes exactly at a certain value of the parameter and the other one is boosted to maximum. This is known as Friedrich-Wintgen BIC [8]. Typically, a pair of eigenmodes M_{mn} and $\mathrm{M}_{m+2,n-2}$ (or M_{mn} and $\mathrm{M}_{m-2,n+2}$) is often used to construct Friedrich-Wintgen BICs. The essence of finding Friedrich-Wintgen BICs is to find two degenerate resonances in a closed resonator with a certain size ratio.

In the present work, we consider the Friedrich-Wintgen BIC in a rectangular resonator embedded in the first channel $p = 1$, provided that other channels are closed for $\nu < 1$. There are numerous degeneracies in a closed rectangular resonator

$$\frac{m^2}{L_x^2} + \frac{n^2}{L_y^2} = \frac{m'^2}{L_x^2} + \frac{n'^2}{L_y^2}. \tag{8}$$

The lowest case corresponds to $m, n = 1, 3$ and $m', n' = 3, 1$ for a square resonator $L_x = L_y$.

After the introduction of the left and right waveguides, these two modes M_{13} and M_{31} are strongly coupled to each other, giving rise to an increase in the destructive interference at a given size ratio. Thus, the resonance frequencies of two modes experience avoided crossing. At the same time, one of the imaginary parts is suppressed to zero while the other is boosted to maximum. Therefore, the formation of such a BIC can be mainly attributed to the destructive interference of modes M_{13} and M_{31} in a closed resonator. We can approximate the eigenfunction of this Friedrich-Wintgen BIC as a superposition of the two eigenmodes of the closed resonator, and its coefficients A and B can be rigorously calculated by

$$\psi_{BIC}(x,y) \approx A\psi_{31}(x,y) + B\psi_{13}(x,y). \tag{9}$$

Substituting Eq. (9) in Eq. (7) gives us

$$A = -W_{1,3;p=1} = -\frac{1}{2\pi}\sqrt{\frac{2L_y}{L_x}}\left[\sin\left(\frac{\pi(Ly+1)}{L_y}\right) - \sin\left(\frac{\pi(Ly-1)}{L_y}\right)\right], \tag{10}$$

$$B = W_{3,1;p=1} = \sqrt{\frac{2}{L_x L_y}}. \tag{11}$$

We can rewrite Eq. (9) as

$$\psi_{BIC}(x, y) \approx \cos\theta\psi_{31}(x, y) + \sin\theta\psi_{13}(x, y), \tag{12}$$

$$\cos\theta = \frac{A}{\sqrt{A^2 + B^2}} \quad , \quad \sin\theta = \frac{B}{\sqrt{A^2 + B^2}} \tag{13}$$

Excellent agreement is found between the eigenfield profile predicted from Eqs.(8-9) and the numerically calculated eigenfield profile of Friedrich-Wintgen BIC, see Fig. S 2.

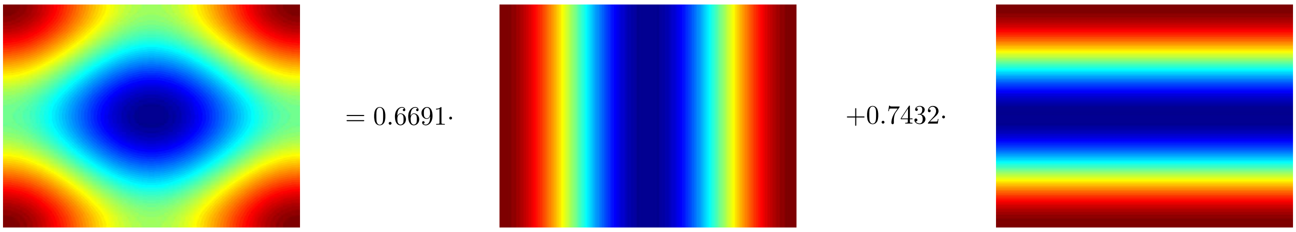

Fig. S 2. Eigenfield profile. Decomposition of Friedrich-Wintgen BIC into eigenmodes M_{31} and M_{13}.

S 2 BIC formation

We investigate a Friedrich-Wintgen BIC induced by mode interference. Therefore, two modes of the same symmetry interact, which results in a highly damped mode and one of increased lifetime. In our configuration, two Friedrich-Wintgen BICs form in a frequency spectrum up to 2300 Hz. The first one at ≈2145 Hz named BIC 1 and the second one at ≈2277 Hz named BIC 2. Both BICs and the corresponding interacting modes are displayed in Figs. S S 3 and S 4.

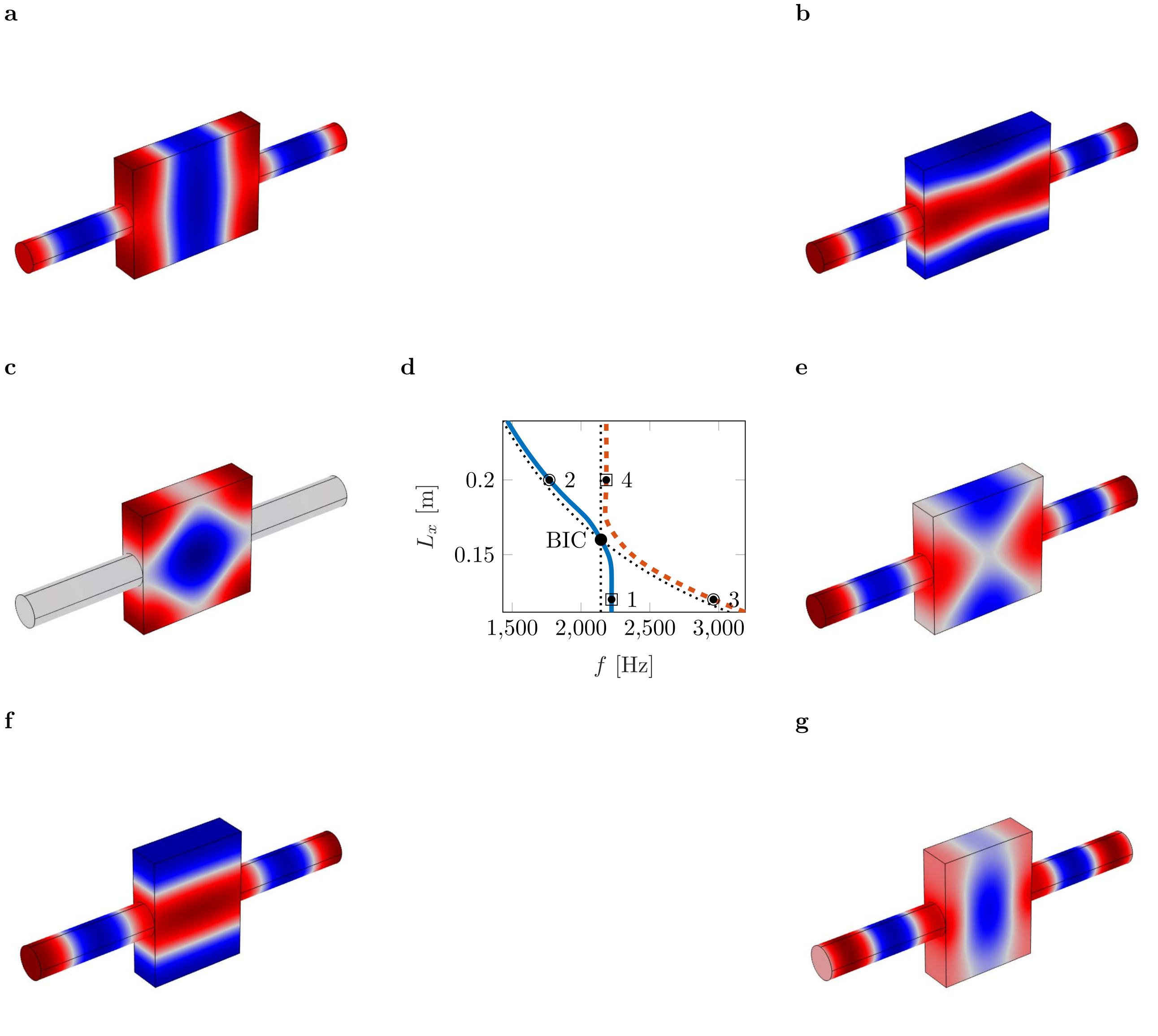

Fig. S 3. Formation BIC 1. a,b,f,g Interacting modes (2,4,1,3) of the same symmetry for different L_x. **c** Mode shape of the BIC. **e** Mode shape of the highly damped mode. **d** Avoided crossing of the eigenfrequencies (real parts) of the interacting modes.

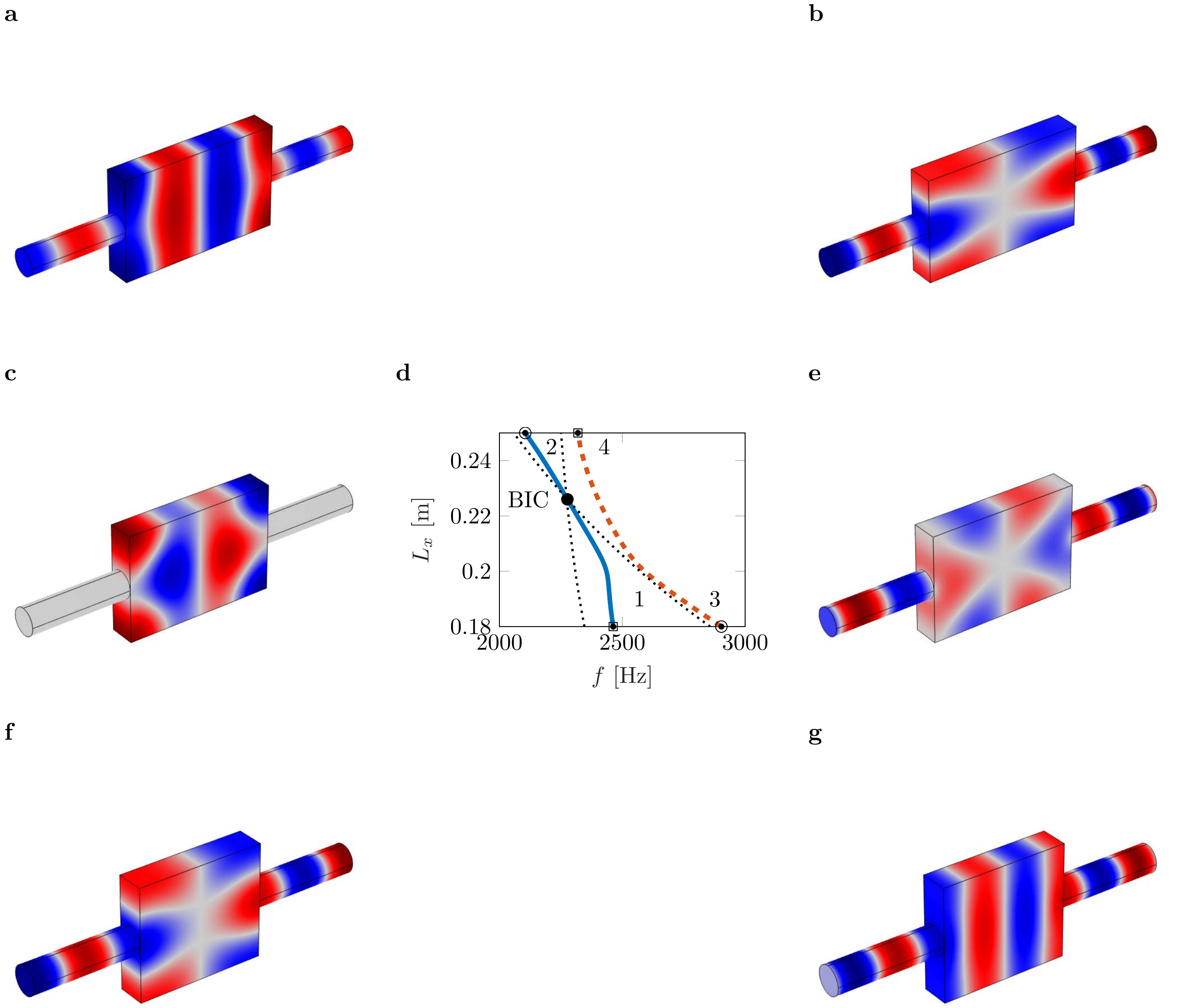

Fig. S 4. Formation BIC 2.. **a,b,f,g** Interacting modes (2,4,1,3) of the same symmetry for different L_x. **c** Mode shape of the BIC. **e** Mode shape of the highly damped mode. **d** Avoided crossing of the eigenfrequencies (real parts) of the interacting modes.

S 3 Complex Eigenfrequencies

The solutions to a waveguide symmetric with respect to the duct axis can be composed of symmetric and antisymmetric solutions [9]. Hence, we can also split the continuum to symmetric and antisymmetric parts. If we excite the waveguide, e.g., by using a background pressure field in the numerical simulations, the symmetric modes can be activated. That means, they couple into the propagating spectrum. The eigenvalues for varying cavity length L_x are shown in Fig. S 5.

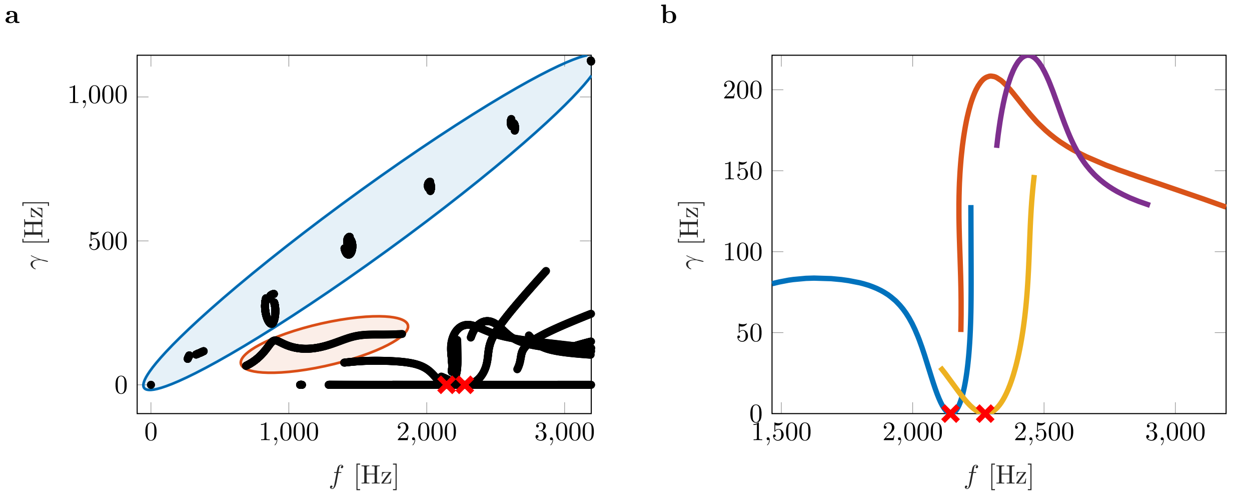

Fig. S 5. Complex eigenfrequencies for varying L_x. a The spectrum of propagating waves dominated by the pipe is marked by the region colored blue. Cavity resonances are highlighted by the region colored red. BIC 1 and BIC 2 are marked by the red crosses. **b** Evolution over L_x of the interacting modes that form BIC 1 & 2.

The blue area is the spectrum of propagating waves dominated by the tube and characterized by high radiation loss. An example of propagating symmetric modes corresponding to cavity resonances are outlined by the red area. Other propagating modes are highlighted by colored curves. Increasing the cavity length results in a lower real part of the eigenfrequencies. Antisymmetric modes cannot radiate away and are therefore localized or trapped modes. They are purely real eigenfrequencies on the x-axis in Fig. S 5. By varying the distance L_x, certain modes interact and their eigenfrequencies pass through an avoided crossing. Therefore, one of the eigenfrequencies has a decreasing imaginary part, becomes purely real and thus a BIC. This is highlighted by the red crosses for BIC 1 and BIC 2.

S 4 Thermo-viscous losses

BICs have a theoretically infinite quality factor. In reality, thermo-viscous losses limit the quality factor to a finite value. We consider losses in our computations. This is done by describing the acoustic boundary layer as a combination of the viscous and thermal boundary layers. Viscous losses occur due to gradients in the velocity field, whereas thermal losses are based on a temperature gradient. We apply the no-slip condition for the velocity field and an isothermal condition for the temperature at the walls of the cavity. Furthermore, we compare the lossless case to the one with losses. The decrease of the Q-factor is displayed in Fig. S 6.

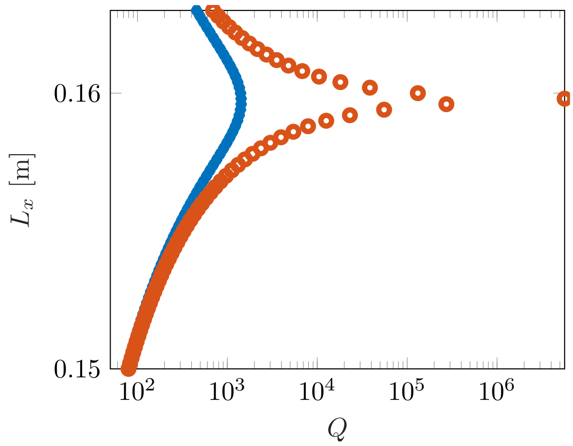

Fig. S 6. Q-factor. Q-factor of the system with (blue line) and without (orange circles) thermo-viscous losses.

We can see a significant reduction of the Q-factor of the lossless case (blue line) compared to one including thermo-viscous losses (orange circles). The effect of thermo-viscous losses on the Fano peaks in the transmission spectra is shown in Fig. S 7.

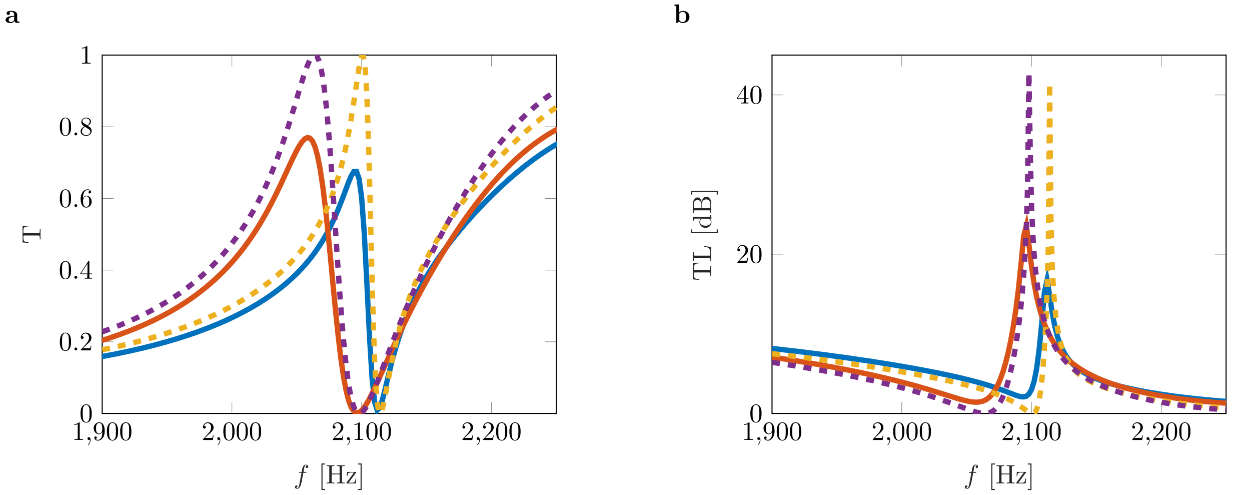

Fig. S 7. Transmission spectra with and without losses. a Transmission coefficient in the 1900 - 2250 Hz frequency range. The solid blue and dashed yellow lines represent the results of $L_x = 165$ mm with and without thermo-viscous losses, respectively. The results of $L_x = 170$ mm are shown by the red and purple lines. **b** Transmission loss of $L_x = 165$ mm and $L_x = 170$ mm with and without losses. The coloring of the lines is identical to Fig. S 7a.

Thermo-viscous losses significantly reduce the transmission coefficient due to increased absorption. Nevertheless, the transmission goes to zero at the frequency of the QBIC, see Fig. S 7a. The maxima of the Fano peaks in the TL also decrease including the losses. We observe a reduction from ≈42 dB to ≈17 dB ($L_x = 165$ mm) and from ≈43 dB to ≈24 dB ($L_x = 170$ mm). The losses have a more significant effect on the amplitude of the

Fano peak of the TL the closer we are to the BIC configuration. In addition, due to thermo-viscous losses, the Fano peaks are shifted to lower frequencies by about 2 Hz. To illustrate the effect of losses on pressure field enhancement, the maximum absolute sound pressure is plotted against cavity length and frequency in Fig. S 8.

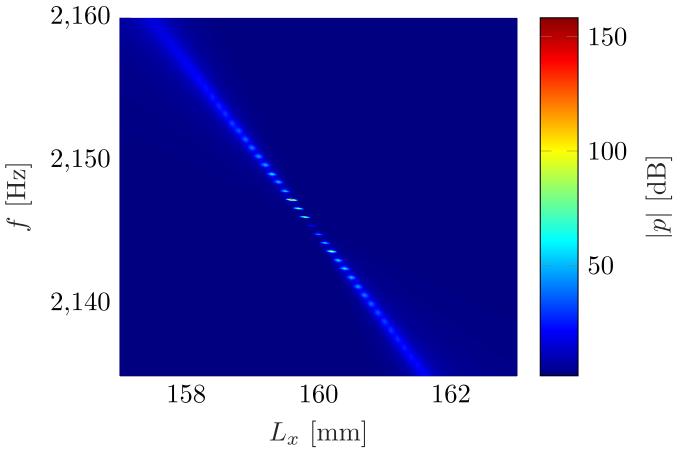

Fig. S 8. Pressure mapping (without losses). Maximum absolute sound pressure inside the rectangular cavity shown in Fig. S 1. The cavity length is varied from L_x = 157 - 163 mm in 0.1 Hz steps in the frequency range 2135 - 2160 Hz.

We observe amplified sound pressure up to 160 dB when excited at 1 Pa and narrow Fano peaks near the BIC. Figs. S 6 to S 8 illustrate the importance of considering thermo-viscous losses in our simulations.

S 5 Analysis of the measured sound pressure field

We use an FFT to further analyze the sound pressure field inside the cavity and thereby obtain the modal coefficients shown in Fig. S 9.

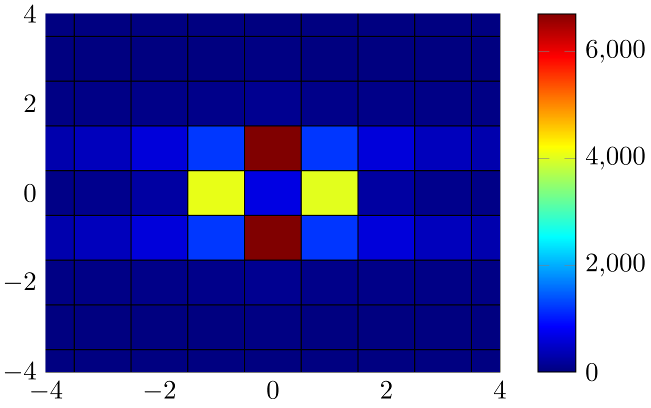

Fig. S 9. FFT coefficients. Coefficients of the fast Fourier transformation corresponding to modes. The indices of the abscissa and the ordinate stand for the modal index in x- and y-direction, respectively. The mode with the indices $(0, 0)$ represents the plane wave.

The four modes with the most dominant Fourier coefficients are the ones that are degenerate with the $(0, 1)$ mode. Hence, the modes with corresponding mode indices $(0, -1), (1, 0), (-1, 0)$. In other terms, referring to Lyapina et al. [2], we denote the modes $(0, 1), (0, -1)$ M_{211} and the modes $(1, 0), (-1, 0)$ M_{121}. A modal superposition by an inverse FFT gives us the sound pressure field of the BIC mode depicted in Fig. S 10a.

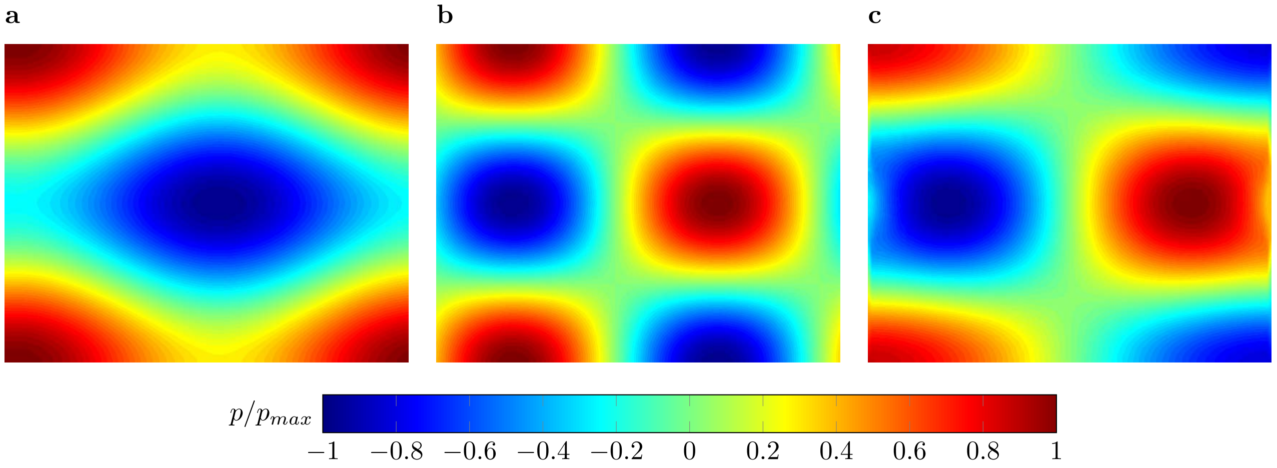

Fig. S 10. Sound pressure fields from modes. **a** Superposition of the four most dominant modes (modes with the highest FFT coefficients). **b** Superposition of the next four modes with high coefficients. **c** Identical mode to the one shown in b obtained from modal analysis. The colored scales display the nondimensonalized pressure, with the pressure being normalized to the maximum pressure of the experiment and the simulation, respectively.

Fig. S 10b shows the sound pressure field of the superposition of the modes that are degenerate with the $(1, 1)$ mode, i.e. the $(1, -1), (-1, 1), (-1, -1)$ modes. The modal analysis of the unexcited system gives us a mode similar the the one in Fig. S 10b, see Fig. S 10c. Therefore, we identify one additional contributing mode excited by the plane wave.

S 6 Pressure enhancement

The configuration with the highest pressure field enhancement is determined by parameter studies. Therefore, we vary the cavity lengths of the configurations depicted in Fig. 1a, Fig. 5a and Fig. 5b. The corresponding plots are shown in Fig. S 11.

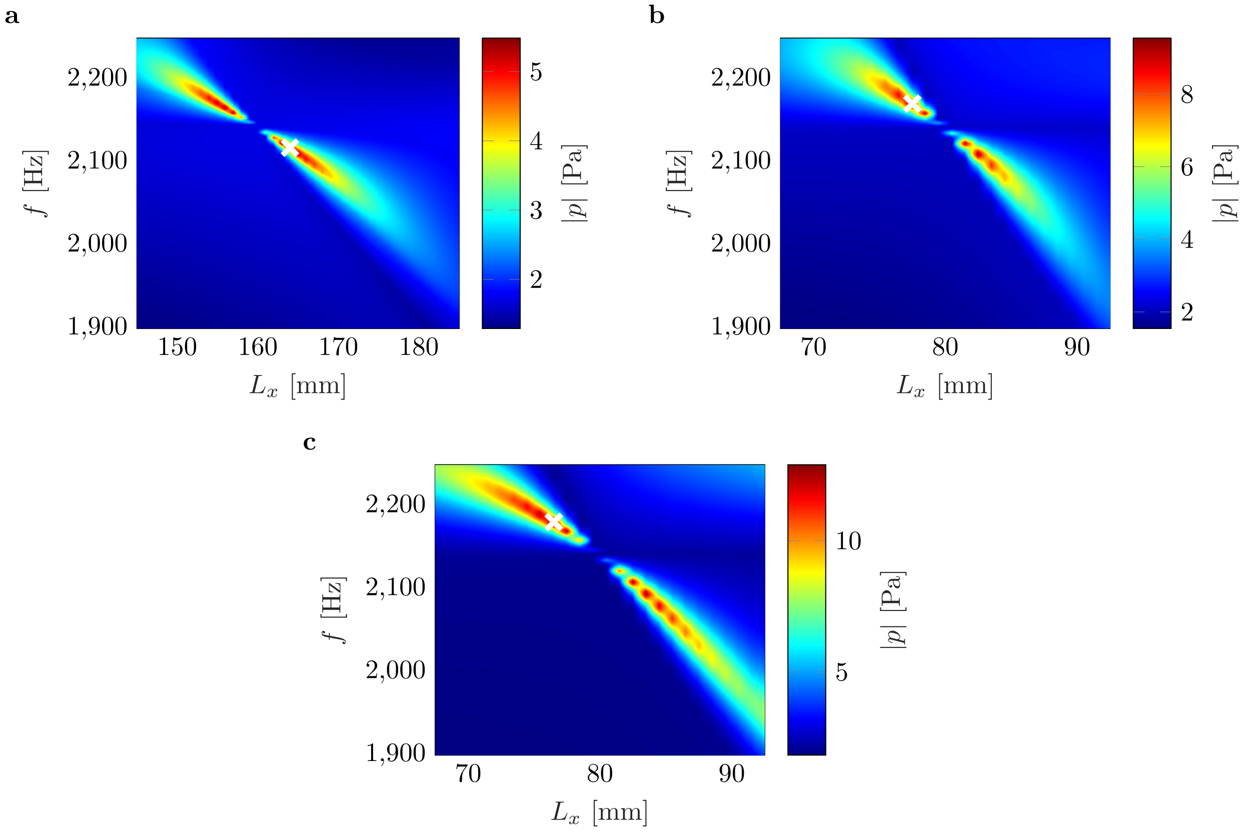

Fig. S 11. Simulated pressure mappings. **a** Maximum absolute sound pressure inside the rectangular cavity shown in Fig. 1a. The cavity length is varied from L_x = 145 - 185 mm in 1 mm and 1 Hz steps in the frequency range 1900 - 2250 Hz. **b** Maximum absolute sound pressure inside the reduced cavity presented in Fig. 5a with cavity length L_x = 67.5 - 92.5 mm in the same frequency range. **c** Maximum absolute sound pressure inside the further reduced cavity (Fig. 5b). The white crosses indicate the maxima.

Pressure enhancement can be observed in certain regions around the BIC configurations. We can also see that the maximum pressure enhancement does not occur directly adjacent to the BIC as it would without losses, i.e., see Fig. S 8. Thermo-viscous losses shift the maximum enhancement away from the BIC configuration. The pressure peaks are 5.50 Pa, 9.54 Pa, and 12.89 Pa. It can be said that the more the geometry is reduced and thus the antisymmetric modes are suppressed, the higher the pressure enhancement. The visualized pressure fields for the full cavity are shown in Fig. S 12.

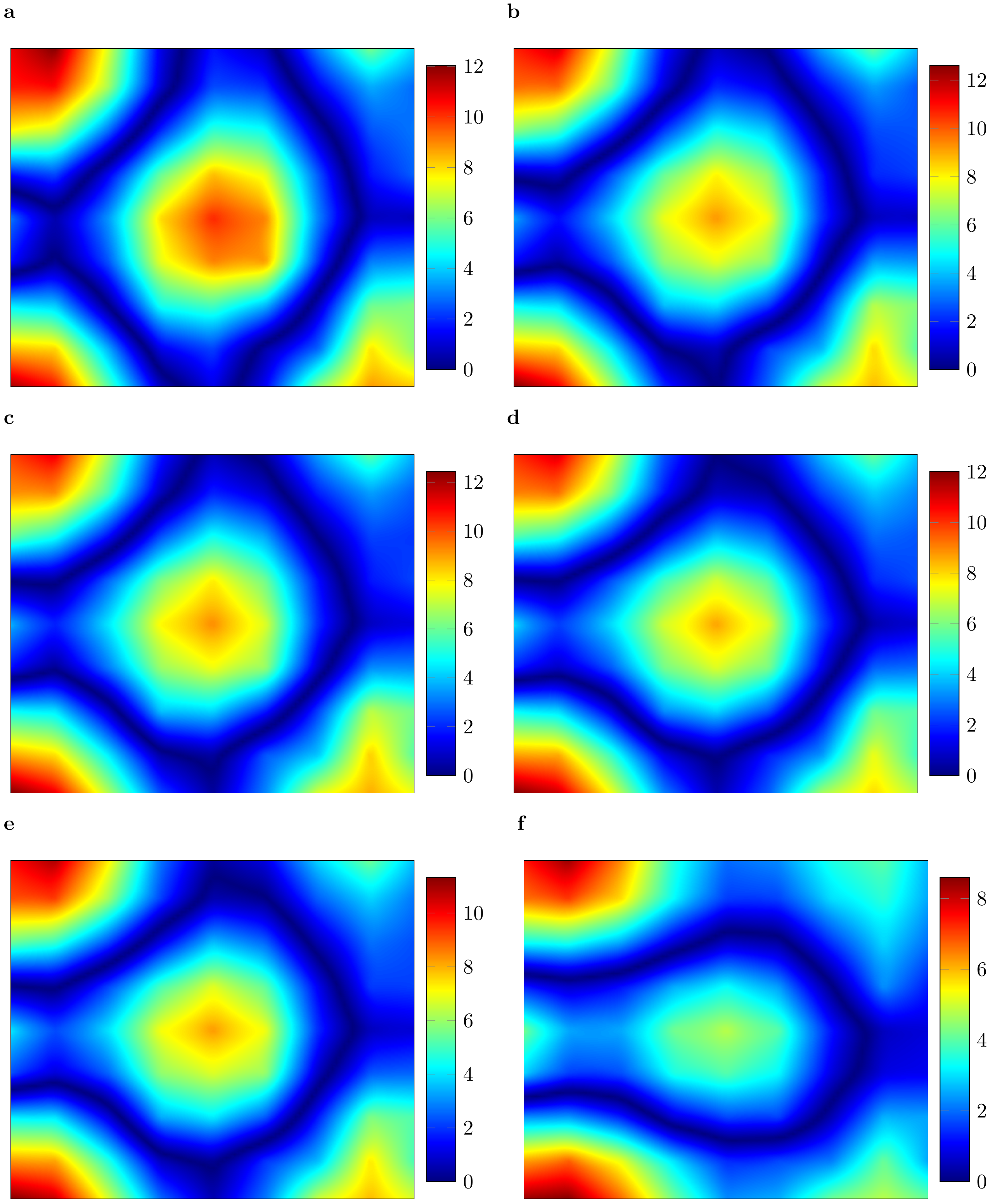

Fig. S 12. Visualized pressure fields of the full cavity. a - f Absolute sound pressure inside the rectangular cavity shown in Fig. 1a. with $L_x = 170\,\mathrm{mm}$ excited at 2070 Hz, 2075 Hz, 2077 Hz, 2079 Hz, 2081 Hz, and 2094 Hz, respectively. All color scales represent the absolute pressure in Pa.

The corresponding maximum pressure values in Figs. S 12a to 12f are 12.04 Pa, 12.61 Pa, 12.44 Pa, 12.02 Pa, 11.32 Pa, and 8.59 Pa, respectively. Therefore, we can say that the maximum pressure enhancement occurs at 2075 Hz. The evolution of the modal field is also demonstrated. The dark blue line representing the pressure

nodes extends in the y-direction, the more the configuration deviates from the BIC configuration.

We numerically determine the configuration with the highest pressure gain by plotting the maximum absolute sound pressure inside the cavity as the cavity length is varied in the 1900 - 2400 Hz frequency range and excited by a plane wave of 1 Pa. This is shown in Fig. S 13 for the fully reduced cavity presented in Fig. 5c.

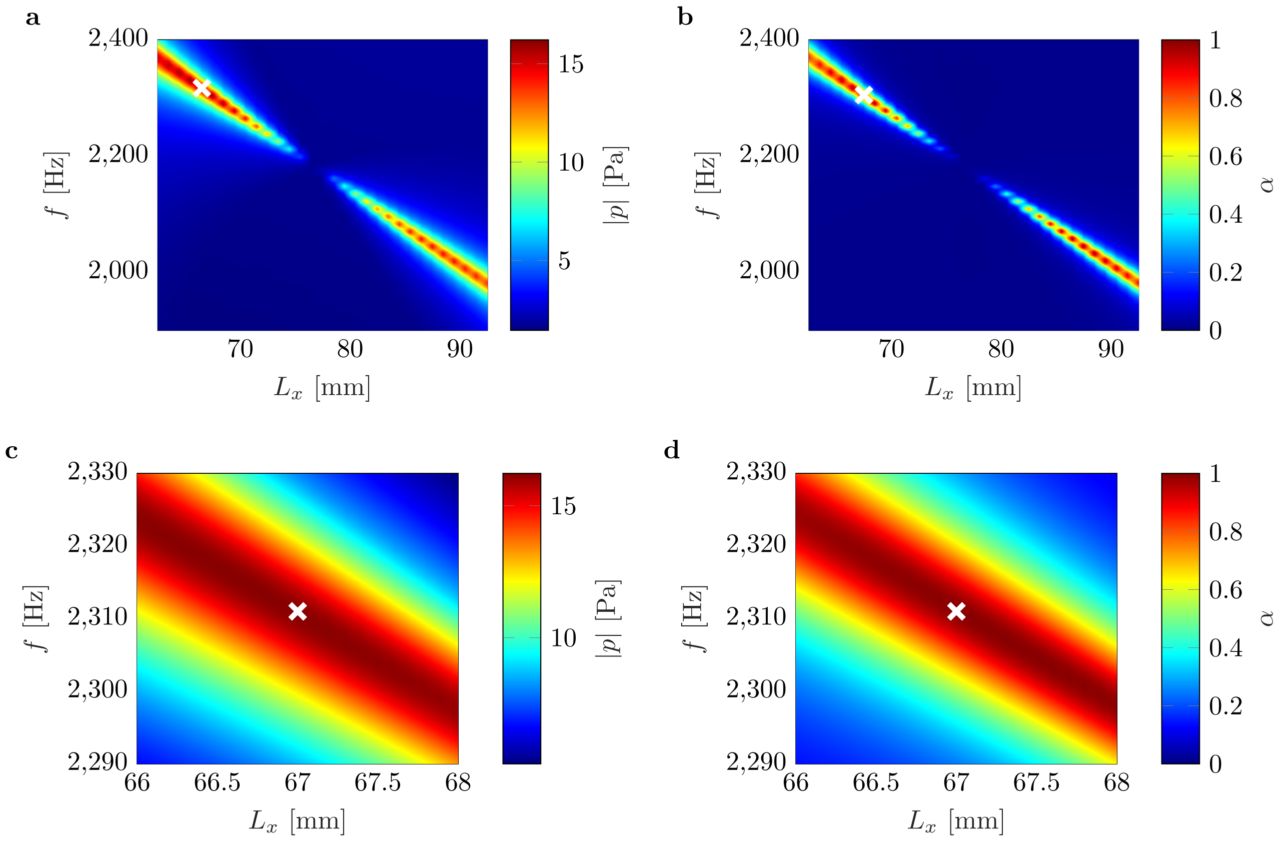

Fig. S 13. Simulated pressure mappings. a,b Maximum absolute sound pressure and absorption inside the cavity shown in Fig. 5c. The cavity length is varied from $L_x = 67.5$ - 92.5 mm in 1 mm and 1 Hz steps in the frequency range 1900 - 2400 Hz. **c,d** Maximum absolute sound pressure and absorption at finer resolution. The cavity length is varied from $L_x = 66$ - 68 mm in 0.1 mm and 0.1 Hz steps in the frequency range 2290 - 2330 Hz. The white crosses indicate the maxima.

The BIC is visible as the dark blue dot in the center of the red lines in Figs. S 13a and 13b. Pressure enhancement can be observed in certain regions around the BIC configurations. The smaller the cavity length, the higher the frequency of the enhancement and vice versa. It can be seen that the maximum pressure enhancement and absorption occurs at a cavity length of $L_x = 67$ mm. We fabricate three additional samples of the fully reduced cavity ($L_x = 64$, 67, and 70 mm) to experimentally validate our numerical predictions, see Fig. S 14.

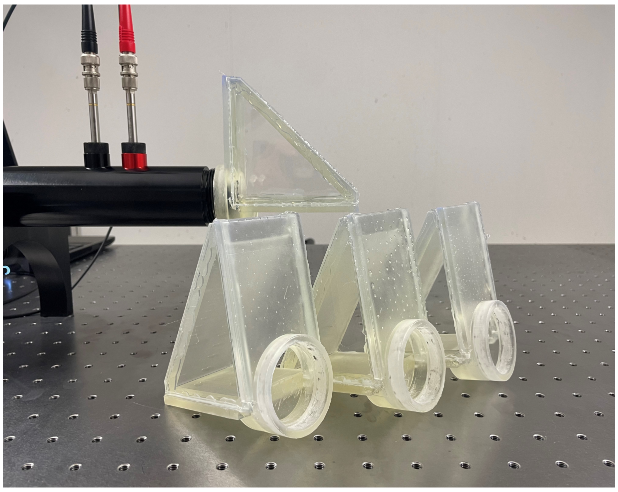

Fig. S 14. LDV samples. Printed samples of the fully reduced cavity with high-transmission glass mounted as side panels.

We then measure the sound pressure field inside the cavity for several frequencies to determine the configuration (frequency and cavity length) where the maximum absolute sound pressure is found. The visualized pressure fields for the fully reduced cavity with $L_x = 64\,\mathrm{mm}$, $L_x = 67\,\mathrm{mm}$, and $L_x = 70\,\mathrm{mm}$ are shown in Fig. S 15.

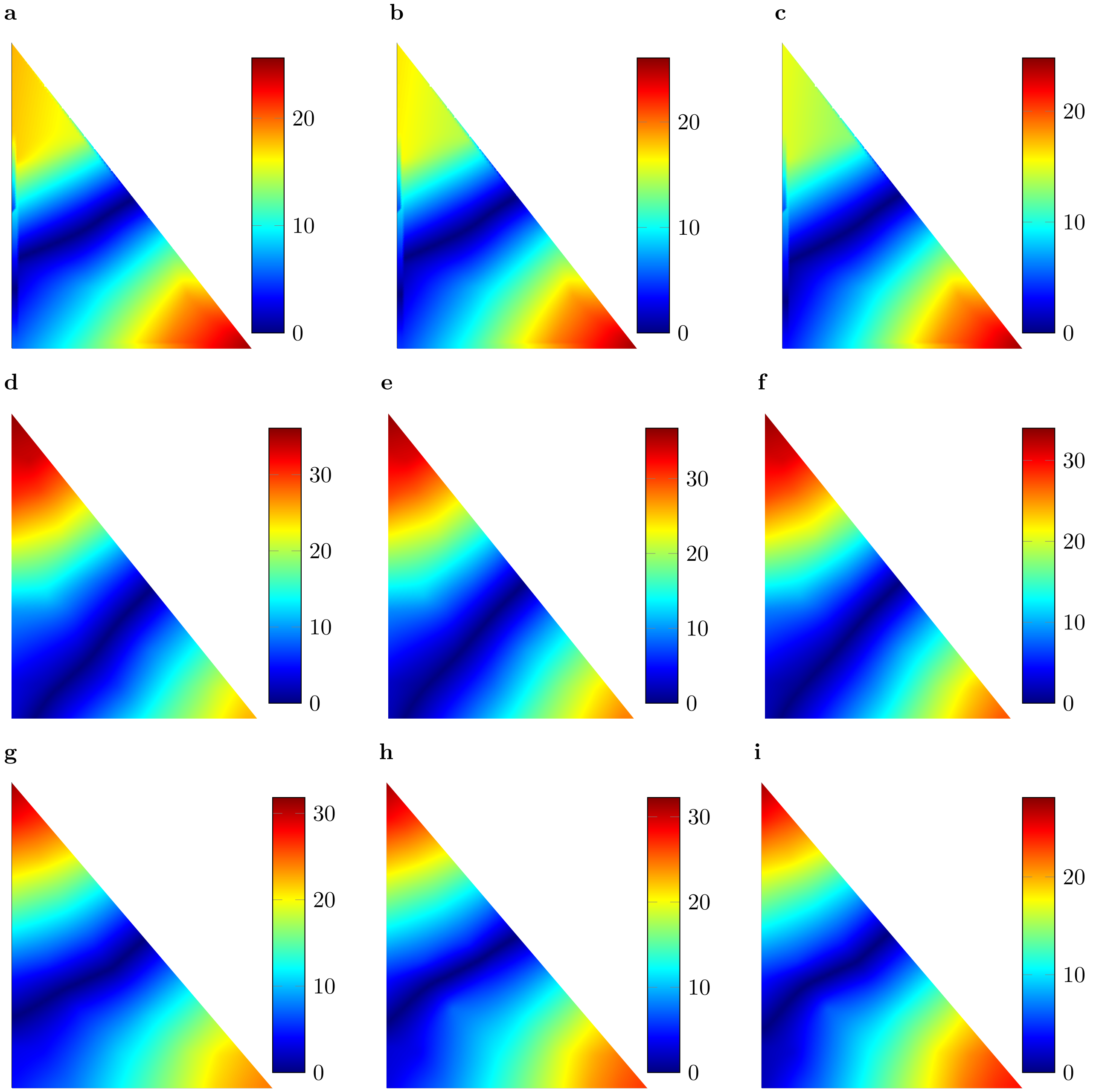

Fig. S 15. Visualized pressure fields of the fully reduced cavity. a - c Absolute sound pressure inside the fully reduced cavity shown in Fig. 5c with $L_x = 64\,\mathrm{mm}$ excited at 2335 Hz, 2345 Hz, and 2353 Hz. **d - f** Absolute sound pressure inside the fully reduced cavity with $L_x = 67\,\mathrm{mm}$ excited at 2310 Hz, 2315 Hz, and 2319 Hz. **g - i** Absolute sound pressure inside the fully reduced cavity with $L_x = 70\,\mathrm{mm}$ excited at 2267 Hz, 2272 Hz, and 2277 Hz. All color scales represent the absolute pressure in Pa.

The data are not normalized to the incident pressure field, but the actual pressure values are shown. The corresponding maximum pressure values in Figs. S 15a to 15i are 25.61 Pa, 26.07 Pa, 24.80 Pa, 36.11 Pa, 36.74 Pa, 33.98 Pa, 31.83 Pa, 32.26 Pa, and 28.12 Pa, respectively. Therefore, we can say that the maximum pressure enhancement for the cavity with $L_x = 64\,\mathrm{mm}$ occurs at 2345 Hz and for the cavity with $L_x = 70\,\mathrm{mm}$ at 2272 Hz. The maximum pressure enhancement occurs at $L_x = 67\,\mathrm{mm}$ at 2315 Hz and reaches 36.74 Pa. This is similar to the results shown in Fig. S 13c.

We compare this maximum pressure value to the sound pressure fields of the fully reduced cavity with $L_x = 64\,\mathrm{mm}$ and $L_x = 70\,\mathrm{mm}$ to prove the existence of a pressure peak. The measurements show peak pressures of 26.07 and 32.26 Pa, respectively. To demonstrate the magnitude of the pressure enhancement, we

also compare it to the pressure field inside the full cavity with $L_x = 170\,\mathrm{mm}$ for several frequencies. The maximum pressure of the full cavity is 12.61 Pa. Thus, the fully reduced cavity leads to the highest pressure enhancement of the investigated Friedrich-Wintgen BIC by a factor of about three.

References

[1] D. Maksimov, A. Sadreev, A. A. Lyapina, and A. Pilipchuk. "Coupled mode theory for acoustic resonators". In: *Wave Motion* 56 (Feb. 2015). DOI: `10.1016/j.wavemoti.2015.02.003`.

[2] A. A. Lyapina, D. Maksimov, A. Pilipchuk, and A. Sadreev. "Bound states in the continuum in open acoustic resonators". In: *Journal of Fluid Mechanics* 780 (June 2015). DOI: `10.1017/jfm.2015.480`.

[3] L. Huang et al. "General Framework of Bound States in the Continuum in an Open Acoustic Resonator". In: *Phys. Rev. Applied* 18 (5 Nov. 2022), p. 054021. DOI: `10.1103/PhysRevApplied.18.054021`. URL: `https://link.aps.org/doi/10.1103/PhysRevApplied.18.054021`.

[4] A. Sadreev. "Interference traps waves in open system: Bound states in the continuum". In: *Reports on Progress in Physics* 84 (Mar. 2021). DOI: `10.1088/1361-6633/abefb9`.

[5] H. Feshbach. "Unified theory of nuclear reactions". In: *Annals of Physics* 5.4 (1958), pp. 357–390. ISSN: 0003-4916. DOI: `https://doi.org/10.1016/0003-4916(58)90007-1`. URL: `https://www.sciencedirect.com/science/article/pii/0003491658900071`.

[6] F.-M. Dittes. "The decay of quantum systems with a small number of open channels". In: *Physics Reports* 339 (Dec. 2000), pp. 215–316. DOI: `10.1016/S0370-1573(00)00065-X`.

[7] J. Okolowicz, M. Płoszajczak, and I. Rotter. "Dynamics of quantum systems embedded in a continuum". In: *Physics Reports* 374 (Feb. 2003), pp. 271–383. DOI: `10.1016/S0370-1573(02)00366-6`.

[8] H. Friedrich and D. Wintgen. "Interfering resonances and bound states in the continuum". In: *Phys. Rev. A* 32 (6 Dec. 1985), pp. 3231–3242. DOI: `10.1103/PhysRevA.32.3231`. URL: `https://link.aps.org/doi/10.1103/PhysRevA.32.3231`.

[9] V. Pagneux. "Trapped Modes and Edge Resonances in Acoustics and Elasticity". In: *Dynamic Localization Phenomena in Elasticity, Acoustics and Electromagnetism*. Ed. by R. V. Craster and J. Kaplunov. Vienna: Springer Vienna, 2013, pp. 181–223. ISBN: 978-3-7091-1619-7. DOI: `10.1007/978-3-7091-1619-7_5`. URL: `https://doi.org/10.1007/978-3-7091-1619-7_5`.
